# Mutation Detection in Tumor-Derived Cell Free DNA Anticipates Progression in a Patient With Metastatic Colorectal Cancer

**DOI:** 10.3389/fonc.2018.00306

**Published:** 2018-08-10

**Authors:** Bruna D. de Figueiredo Barros, Bruna E. C. Kupper, Samuel Aguiar Junior, Celso A. L. de Mello, Maria D. Begnami, Rubens Chojniak, Sandro J. de Souza, Giovana T. Torrezan, Dirce M. Carraro

**Affiliations:** ^1^Laboratory of Genomics and Molecular Biology—International Research Center/CIPE, A. C. Camargo Cancer Center, São Paulo, Brazil; ^2^Colorectal Tumors Department, A. C. Camargo Cancer Center, São Paulo, Brazil; ^3^Clinical Oncology Department, A. C. Camargo Cancer Center, São Paulo, Brazil; ^4^Department of Anatomic Pathology, A. C. Camargo Cancer Center, São Paulo, Brazil; ^5^Imaging Department, A. C. Camargo Cancer Center, São Paulo, Brazil; ^6^Bioinformatics Multidisciplinary Environment, Digital Metropolis Institute, Federal University of Rio Grande do Norte, Natal, Brazil; ^7^Brain Institute, Federal University of Rio Grande do Norte, Natal, Brazil

**Keywords:** liquid biopsy, ctDNA, colorectal cancer, NGS, gene panel

## Abstract

**Background:** The observation of tumor-derived cell-free DNA (ctDNA) in plasma brought new expectations to monitor treatment response in cancer patients.

**Case presentation:** In an exploratory case of a 57-year-old man diagnosed with metastatic sigmoid adenocarcinoma, we used a hotspot panel of cancer-associated gene mutations to identify tumor-specific mutations in the primary tumor and metastasis. Results: Five mutations were detected (*KRAS*, p.Gly12Val; *TP53*, p.Arg175His; *RB1*, p.Ile680Thr; *ALK*, p.Gly1184Glu; and *ERBB2*, p.Lys860Lys), of which three were detected in both tissue types (primary tumor and metastasis). All five mutations were monitored in the ctDNA of six serial plasma samples. Only *KRAS* and *TP53* mutations were detected at a high frequency in the first plasma sample. After 1 month of chemotherapy the allele frequencies of both mutations fell below the detection limit. From the third month of systemic treatment onward, the allele frequencies of both mutations were detectable in plasma, displaying a continual increase thereafter. The remaining three mutations were not detected in plasma samples. Signs of disease progression in ctDNA during the treatment period were evident while computed tomography (CT) measurements suggested stable metastatic lesions throughout the treatment.

**Conclusions:** Liquid biopsies revealed tumor heterogeneity and predicted tumor progression, demonstrating the potential of ctDNA analysis to be a sensitive and specific tool for monitoring treatment responsivity and for early identification of treatment resistance.

## Background

Recently, there have been substantial advances in the development of minimally invasive techniques for cancer diagnostics and the monitoring of treatment response and disease progression. In this context, liquid biopsies are emerging as a valuable tool in several clinical scenarios.

Liquid biopsies can be use to detect tumor biomarkers, such as circulating tumor cells, tumor microvesicles and circulating tumor DNA (ctDNA) in plasma and other body fluids. One of the most validated liquid biopsies applications is the detection of tumor-specific mutations in cell-free DNA (cfDNA) that has been released into circulation. Owing to the non-invasive nature of liquid biopsies making them amenable to repeat procedures, ctDNA detection in liquid biopsies has been demonstrated to be useful for several oncological applications, including monitoring treatment response, disease progression, and tumor relapse information (1–6). Additionally, this method enables assessment of tumor variability and heterogeneity, once tumor DNA are expelled from cells from different regions of the tumor and metastases (3, 7, 8).

Until recently, detection of ctDNA was hindered by the insufficient sensitivity of routine techniques for assessing the tumor-specific mutations in total cfDNA. Because cfDNA contains DNA from both normal and cancer cells, with the latter being found at lower levels and in much more degraded fragments, highly sensitive techniques are needed to detect tumor-specific mutations (3, 6, 9, 10).

A great number of variables can also limit cfDNA concentrations and hinder ctDNA detection. For example, trauma, infection, autoimmune disease, and intensive exercise, can alter cfDNA concentrations in plasma (11, 12). Furthermore, because ctDNA has a half-life is under 2.5 h, the time elapsed between sample collection and processing can reduce the ctDNA detection potential (13, 14). Moreover, ctDNA levels appear to be associated with tumor burden, such that more advanced tumors are more likely to produce higher amounts of ctDNA in plasma (6), whereas a favorable treatment response can decrease tumor DNA quantities in circulation (13, 15). Another important issue is the clonal hematopoiesis of indeterminate potential, commonly referred to as CHIP. Several studies have demonstrated that aging can increase somatic mutations in cfDNA of healthy individuals, especially in genes associated with hematopoietic elements, such as *DNMT3A* and *TET2* (16–18). Thus, it is important to take precautions with respect to these variables to avoid erroneous diagnostic conclusions.

To obtain the sensitivity and specificity necessary to incorporate ctDNA analysis into clinical practice, existing techniques may be modified and/or new techniques may be developed. To date, the most used technologies have been digital PCR, real-time PCR, BEAMing (beads, emulsions, amplification, and magnetics) (19, 20), and, most recently, next generation sequencing (NGS).

NGS has been shown to be a reliable tool for detecting tumor-specific mutations in ctDNA with great sensitivity and specificity (>94%, and >98%, respectively) (5, 6). Changes in ctDNA levels corresponding to tumor dynamics in response to treatment have been demonstrated across several tumor types, including lung, breast, colorectal, and melanoma cancers among others (2, 4, 6, 14, 21–23). Increases in ctDNA mutation allele frequencies were shown to occur prior to clinical or imaging evidences of tumor progression (24). Because treatment resistance can result from the acquisition of new somatic mutations in cancer cells, genomic ctDNA profiling may enable detection of emerging subclonal actionable mutations for which targeted therapies can be applied.

Here, we examined the relevance of ctDNA analysis in a tumor kinetics study involving a patient diagnosed with metastatic colorectal cancer using a NGS hotspot panel of 50 genes frequently mutated in cancer. We evaluated both primary and metastatic tumor tissues and monitored ctDNA extracted from plasma samples collected over the course of patient's treatment.

## Case presentation

A 57-year-old man presented with a complaint of increasing abdominal pain in June of 2014. A colonoscopy performed in July of the same year showed a stenotic and ulcerated lesion with an infiltrative aspect in the sigmoid region; the stenosis prevented advancement of the colonoscopy beyond the lesion. Computed tomography (CT) revealed hepatic nodules with peripheral contrast enhancement in segments II, IV, I, VIII, V, and VI. The largest hepatic nodule measuring 2.5 cm, was found in segment II. Additionally, a hypodense nodular formation, measuring 2.8 cm, was found in the right adrenal gland and a focal wall thickening, with an area of 6.0 × 3.2 cm, was found in the descending colon measuring. A subsequent magnetic resonance imaging (MRI) examination conducted in August of 2014 revealed hepatic nodules larger than 4.4 cm in segment I. A thoracic CT performed on the same date showed pulmonary micronodules suggestive of secondary implants.

Due to the obstructive sigmoid lesion, a laparoscopic sigmoidectomy with primary colorectal anastomosis was considered the first treatment option, followed by palliative chemotherapy. The patient received FOLFOX (10 cycles) as a first-line treatment and FOLFIRI (3 cycles) as second-line regimen. Further evaluations of the hepatic lesions were made every 2–3 months by CT imaging. Carcinoembryonic antigen (CEA), a serum marker used to monitor carcinoma progression, was evaluated at the time of diagnosis (145 mg/dl) and before commencement of the second-line treatment (1,678 mg/dl).

Microscopic evaluation of the surgical specimen revealed a moderately differentiated sigmoid adenocarcinoma (5.6 × 3.4 cm) with mucinous pattern areas and a pathology stage of pT4apN2apM1. The lesion had an invasive front compromising the serous layer. Lymph-node metastases with capsular extravasation were detected in four of fourteen lymph-nodes dissected from adjacent adipose. Surgical margins were tumor-free. Biopsies of hepatic growths at the moment of the primary tumor surgery confirmed a diagnosis of metastatic colorectal adenocarcinoma. Immunohistochemistry showed positive labeling for the mismatch repair proteins MLH1, MSH2, MSH6, and PMS2. An activating *KRAS* mutation was identified by routine molecular testing for metastatic colorectal cancer at our institution.

Targeted resequencing was performed in the Ion Proton platform with the Ion AmpliSeq™ Cancer Hotspot Panel v2 (Thermo Fisher Scientific, Waltham, MA), which covers approximately 2,800 COSMIC mutations from 50 oncogenes and tumor suppressor genes. A detailed description of the sequencing methods are provided in a Supplementary File. Tumor-specific genomic DNA mutations were assessed for the primary tumor, and metastasis. cfDNA from six plasma samples (PS1–6) were also assessed: one before treatment and five after surgery and during palliative chemotherapy.

Sequencing analysis of the primary tumor identified five tumor-specific mutations, including an activating *KRAS* mutation (p.Gly12Val), confirming previous analysis, a loss-of-function mutation in *TP53* (p.Arg175His), two somatic mutations of unknown clinical impact in *RB1* (p.Ile680Thr) and *ALK* (p.Gly1184Glu), and a synonymous *ERBB2* (p.Lys860Lys) alteration (Table 1). Evaluation of hepatic metastatic lesions detected three of these somatic mutations (*KRAS, TP53*, and *RB1*) with a 1% threshold criterion. In the plasma sample collected before surgery (PS1), only the two mutations (*KRAS* and *TP53*) detected with high allele frequency in both primary and metastatic samples were detected in ctDNA.

**Table 1:** Somatic mutations identified in the primary tumor, metastasis and plasma.

**Gene**	**Chr: position**	**Ref allele**	**Mut allele**	**Codon change**	**Protein change**	**Variant type**	**VAF%**		
							**Tumor**	**Metastasis**	**PS1**
*KRAS*	chr12: 25398284	C	A	c.35G>T	p.Gly12Val	Missense	43.28	35.86	23.23
*TP53*	chr17: 7578406	C	T	c.524G>A	p.Arg175His	Missense	80.23	61.35	28.27
*RB1*	chr13: 49033902	T	C	c.2039T>C	p.Ile680Thr	Missense	11.11	1.08	ND
*ALK*	chr2: 29443666	C	T	c.3551G>A	p.Gly1184Glu	Missense	25.89	ND	ND
*ERBB2*	chr17: 37881388	A	G	c.2580A>G	p.Lys860Lys	Synonymous	19.66	ND	ND

*Chr, chromosome; Ref, reference allele; Mut, mutated allele; VAF, variant allele frequency; PS1, plasma sample #1 (pretreatment) ND, not detected*.

**Figure 1:**
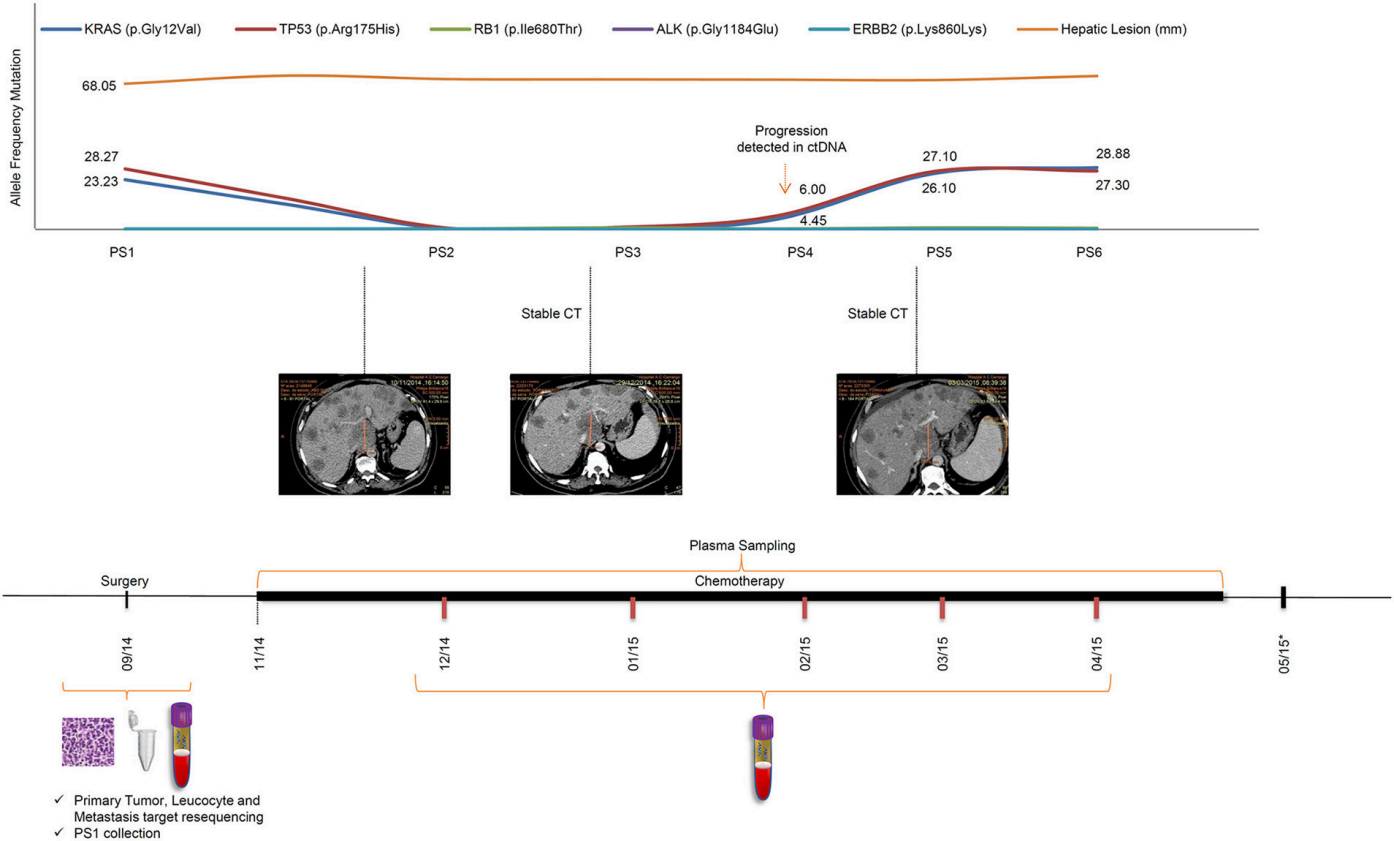
Timeline of patient's treatment, follow-up and ctDNA mutation levels during disease progression. The upper panel shows the frequencies of tumor mutations detected in each plasma sample, and hepatic lesion size throughout the course of palliative chemotherapy. An increase in *KRAS* and *TP53* mutation frequencies observed in PS4 preceded significant increases in tumor marker frequencies in PS5 and PS6. Sequential CT measurements were suggestive of disease stability throughout the treatment period. The lower panel shows the patient's course of treatment (FOLFOX followed by FOLFIRI) with surgery date and serial PS collection. PS, plasma sample; CT, computed tomography; *, patient death.

The remaining five additional plasma samples, PS2–6, were collected monthly, starting 1 month after the beginning of chemotherapy (Figure 1). Interestingly, the allele frequency of the two aforementioned tumor-specific mutations in *KRAS* and *TP53* decreased significantly, dropping below the 1% detection cut-off of by PS2 (Figure 1). ctDNA mutations remained undetectable by NGS in PS3. The noticeable decrease in allele frequency mutations after primary tumor resection and during palliative chemotherapy can be related to a response to treatment. By PS4, in February of 2015, 3 months of FOLFOX treatment, the allele frequencies of both mutations started to rise, *TP53* 6% and *KRAS* 4%, approaching PS1 frequencies in PS5 and PS6. Although the initial decrease in mutation frequencies was in accord with an initial treatment response; subsequent increases in mutation frequencies anticipated tumor progression, albeit CT imaging showed maintenance of the number and size of the patient's liver lesions throughout palliative chemotherapy treatment. The timeline of the patient's peripheral blood collection, palliative chemotherapy, and follow-up scheme, as well as the ctDNA identification through NGS, along with their frequencies, are shown in Figure 1. In April of 2015, the patient presented signs of disease progression and FOLFIRI was started. After 3 cycles of FOLFIRI, the patient's clinical condition deteriorated, and he died due to liver failure in May of 2015. It is important to highlight that ctDNA analysis was not performed concurrently with plasma collection, and all ctDNA analysis were performed at a later time, such that ctDNA results did not alter the clinical treatment protocol.

## Discussion

In this case study we were able to detect five tumor-specific mutations in primary or metastatic tumor tissues and use them to monitor tumor dynamics in plasma samples. Two mutations that are strongly associated with colorectal cancer (*KRAS*, and *TP53*) exhibited robustly elevated allele frequencies in the preoperative plasma sample. The allele frequencies of both mutations decreased sharply after resection of primary tumor and continued decreasing in the first month of chemotherapy, suggesting that the metastatic lesions were responding to the treatment. The subsequent increasing trend in these mutated allele frequencies were suggestive of tumor progression. Interestingly, all CT evaluations performed in this timeframe suggested disease stability, with no evidence of increase in the number or size of the major lesions. Disease progression was detected in an MRI exam (data not shown) performed 24 days after the last CT; showing innumerous diffuse and confluent liver metastases interpreted as unequivocal disease progression. Although MRI has a higher sensitivity than CT, the tumor burden change observed was significant and would probably have been detected by CT before patient's death. Nevertheless, we cannot infer neither that these further lesions were not present earlier nor that MRI could have detected tumor progression if used for continuous monitoring of the patient. Notwithstanding, the early change in the dynamics of the ctDNA was predictive of a poor outcome, as shown in our ctDNA analysis timeline.

Morphological imaging-based criteria are still the main parameter utilized to monitor solid tumor evolution, but have limitations. A critical drawback is the delay between tumor progression/regression and a perceptible change in tumor size. Detecting tumor enlargement by imaging can take weeks or months, which can delay critical decisions in patient management. The present data support this conclusion.

Several evidences have indicated that ctDNA frequencies are associated with cancer patient prognosis and can anticipate disease progression, in a manner that is more precise than the methods currently used for monitoring chemotherapy response. In the present case, CEA, a tumor marker use to monitor treatment response in patients with colorectal cancer patients, did also show concordance with clinical progression, however, several limitations have been recurrently reported regarding this issue. About 30% of patients diagnostic with colorectal cancer do not show alterations in CEA levels (25); not every patient has abnormal CEA elevation in the presence of disease and during relapse; CEA flare post and during chemotherapy are not always related to cancer progression; among others.

Similar to our results, Bettegowda et al. observed a correlation between elevations in ctDNA frequency and poor prognosis and overall survival in various malignancies. Moreover, Dawson et al. (15) observed increased ctDNA frequencies in a group of women with metastatic breast cancer, earlier to imaging exams. Thus, ctDNA seems to be a trustworthy biomarker when compared to the current ones used in diagnosis including imaging diagnostic methods.

NGS provides a window into tumor dynamics and resistance mechanisms by providing data for a larger number of mutations. The information obtained is of an inestimable value because it may enable early detection of disease progression, which may affect the planning for combination treatments and the use of alternate therapies, thereby providing the potential to hasten and improve disease management decisions (9, 10, 15, 26). Using a 50-gene panel for screening the primary tumor, metastatic and plasma samples, we were able to assess the heterogeneity among them and the clonal dynamics of tumor cells in response to treatment.

It is noteworthy that we identified only *KRAS* and *TP53* mutations in ctDNA. Activating mutation in *KRAS* and loss-of-function mutation in *TP53* seem to be essential for tumor maintenance and progression (27). The *KRAS* activating mutation p.Gly12Val results in constitutive activation of RAS GTPase and is considered to be a driver mutation in colorectal cancer (28). Meanwhile, *TP53* p.Arg175His, a known pathogenic mutation, has been observed in 6% of colorectal cancers and 3% of head and neck cancers [(29); COSMIC database]. Our data reinforce the notion that investigating a group of tumor mutations enables a broad assessment of tumor mutation burden dynamism during treatment and disease progression.

Another interesting finding of our study was the fact that not all mutations could be tracked by ctDNA analysis. Initially, we found five point mutations in the primary tumor, some with potential clinical relevance (*ALK* and *ERBB2*). However, metastasis sequencing revealed only three mutations *KRAS* and *TP53*, at high-level, and *RB1*, at low-level. None of the additional mutations (*ALK, ERBB2*, and *RB1*) were detected in the plasma samples.

The RECIST imaging criteria is used universally to evaluate tumor response during and after systemic treatment for solid tumors. This criteria, which is related to tumor size changes, strongly correlates with clinical outcome (progression, symptoms, and death). However, it is becoming increasingly clear that other methods not based solely on tumor size should be developed and validated to improve our ability to evaluate treatment efficacy in oncology. The liquid biopsy technique represents one promising option. In the present case, despite no tumor alterations was detected on CT after treatment, we observed early decreases and subsequent increases in ctDNA. In a scenario where multiple and personalized drugs are available, this strategy may inform rapid changes in an ongoing treatment plan.

## Conclusions

Our results reinforce the potential applicability of plasma ctDNA for anticipating disease progression efficiently in patients with colorectal cancer and highlight the value of NGS in revealing clonal dynamics of tumors in response to therapy.

## Ethics statement

Patient has signed an informed consent form to participate in the study and for case report publication. This study was performed in compliance with the Helsinki Declaration and was approved by the ethics committee of the A. C. Camargo Cancer Center under number 1819/13.

## Author contributions

BDFB carried out the sequencing experiments. BK was responsible for the patient's follow up. RC evaluated CT imaging results. CALM, SAJ, SJS, and DMC designed the study. BDFB, GTT, CALM, RC and DMC analyzed the data and wrote the manuscript. All authors contributed to manuscript revision and read and approved the submitted version.

### Conflict of interest statement

The authors declare that the research was conducted in the absence of any commercial or financial relationships that could be construed as a potential conflict of interest.

## Abbreviations

CEA: carcinoembryonic antigen
ctDNA: circulating tumor DNA
CT: Computed tomography
cfDNA: cell free DNA
NGS: Next Generation Sequencing
CHIP: Clonal hematopoiesis of indeterminate potential
COSMIC: Catalog of Somatic Mutations in Cancer
MRI: magnetic resonance imaging
PS: Plasma sampling
RECIST: Response Evaluation Criteria In Solid Tumors.
