# Supplementary material for: Mutation Detection in Tumor-Derived Cell Free DNA Anticipates Progression in a Patient With Metastatic Colorectal Cancer
Source: Front Oncol. 2018 Aug 10;8:306. doi: 10.3389/fonc.2018.00306 (PMC6095987; doi:10.3389/fonc.2018.00306)
Supplement: Supplementary file 1 [file Table_1.DOCX]

Supplementary Material

**Mutation detection in tumor-derived cell free DNA anticipates progression in a patient with metastatic colorectal cancer**

Bruna Durães de Figueiredo Barros, Bruna Elisa Catin Kupper, Samuel Aguiar Junior, Celso Abdon Lopes de Mello, Maria Dirlei Begnami, Rubens Chojniak, Sandro José de Souza, Giovana Tardin Torrezan, Dirce Maria Carraro*

*** Correspondence:** Corresponding Author: [dirce.carraro@accamargo.org.br](mailto:dirce.carraro@accamargo.org.br)

# Supplementary Data

Materials and Methods

**Casuistic**

Fresh frozen tissue, and blood sample from a patient with metastatic sporadic colorectal adenocarcinoma from AC Camargo Cancer Center was used for identification of somatic mutations, and mutation screening in plasma samples. Samples were obtained in accordance with the authorization of the AC Camargo Cancer Center Ethics Committee.

**DNA Extraction**

Fresh frozen tumor and normal samples were submitted to a second histological analysis by a pathologist in order to ensure representative tumor selection. Histological analysis of the tumor showed adenocarcinoma in 100% of the slide, metastasis of the adenocarcinoma also presented tumor cells in 100% of the slide, normal tissue had non-neoplastic colonic mucosa in 100% of the histological slide. Genomic DNA of all tissues was extracted using a standard proteinase K-phenol-chloroform protocol design by the AC Camargo Biobank.

Blood samples were processed within 2 hours of collection at the Macromolecule’s Bank of AC Camargo Cancer Center to preserve DNA integrity. Serial samples of peripheral blood (4ml) were collected in BD Vacutainer®/HemogardTM EDTA K2 Plus tubes (BD Biosciences, New Jersey, EUA) and submitted to centrifugation at 200g for 10 minutes, followed by a 10 minutes centrifugation at 1.600g at room temperature.

The plasma was transferred to a new tube and submitted to centrifugation at 1.600g for 10 minutes, in order to remove remaining leukocytes. After this step, plasma sample was purified using the QIAamp Blood Midi Kit (QIAGEN, Germany), according to the manufacturer’s instructions.

The remaining total blood cells were lysed in 1X Tris-EDTA buffer pH8,0, followed by centrifugation at 3.500rpm for 5 minutes to separate leukocytes. Genomic DNA from leukocyte pellet was purified using the QIASymphony DNA Midi Kit at the QIASymphony equipment (QIAGEN, Hilden, Germany), according to the manufacturer’s instructions.

DNA purity of all samples was assessed using NanoDrop ND-1000 spectrophotometer (Thermo Scientific, Wilmington, Delaware, USA), concentration with Qubit® 3.0 Fluorometer (Thermo Scientific, Wilmington, Delaware, USA), and integrity by electrophoresis in an agarose gel and visualization with SYBR-Safe DNA gel stain (10,000X concentrated in DMSO) (Thermo Scientific, Wilmington, Delaware, USA). All samples were processed at the AC Camargo Biobank.

**Somatic Mutations Identification**

In order to identify somatic mutations, we used a target-sequencing strategy using the Ion Ampliseq™ Cancer HotSpot Panel V2 (Thermo Fisher Scientific, Waltham, Massachusetts, EUA) (which covers approximately 2,800 COSMIC mutations from 50 oncogenes and tumor suppressor genes), in primary tumor, metastasis and leucocyte. Samples were prepared using 10ng of genomic DNA accordingly to manufacturer’s instructions. Template preparation, emulsion PCR, Ion Sphere Particles enrichment and high-throughput sequencing were performed using Ion Proton platform, and sequencing run was performed using Ion PI™ Chip according to the manufacturer’s instructions (Thermo Fisher Scientific, Waltham, Massachusetts, EUA).

The reads obtained after sequencing were quality-filtered and sorted according to barcodes through the sequencer software (Torrent Suite Browser 5.0.3). The sequences obtained were analyzed using the VariantCaller (tvc 5.0-6) plugin from Torrent Suite Browser. Parameters used to call alterations were: minimum allele frequency of 0.02 for SNP and INDEL; minimum quality of 10; minimum coverage of 20; minimum coverage in each strand of 3; maximum strand bias of 0.9 for SNP and 0.85 for INDEL. VCF file was used to select variants to screen in plasma samples.

For somatic mutations, alterations were considered if: i) minimum coverage depth of 100x in both primary tumor and leucocyte, ii) mutation frequency in tumor higher than 10% and not identified in leucocyte, iii) at least 4 reads with the alternated base. To screen for tumor-specific mutations in plasma samples, we established a minimum coverage of 1000x if we identified the mutation, and a minimum coverage of 10.000x of the alternate base to assert absence of mutation. For mutation screening in cfDNA six plasma samples were assessed - one before and five after surgery and during palliative chemotherapy.

Even if the alternate base from tumor-specific mutation was not called in any of the plasma sample, visual evaluation of the BAM files was performed using CLCBio Workbench (Aarhus, Denmark). Sequencing raw data is available under request.
